# Helicity Enhanced Torsion Sensor Based on Liquid Filled Twisted Photonic Crystal Fibers

**DOI:** 10.3390/s20051490

**Published:** 2020-03-09

**Authors:** Feng Zhang, Ying Wang, Zhiyong Bai, Shen Liu, Cailing Fu, Yijian Huang, Changrui Liao, Yiping Wang

**Affiliations:** 1 Key Laboratory of Optoelectronic Devices and Systems of Ministry of Education and Guangdong Province, College of Optoelectronic Engineering, Shenzhen University, Shenzhen 518060, China; zhangfeng@szu.edu.cn (F.Z.); baizhiyong@szu.edu.cn (Z.B.); shenliu@szu.edu.cn (S.L.); fucailing@szu.edu.cn (C.F.); huangyijian@email.szu.edu.cn (Y.H.); cliao@szu.edu.cn (C.L.);; 2 Guangdong and Hong Kong Joint Research Centre for Optical Fibre Sensors, Shenzhen University, Shenzhen 518060, China

**Keywords:** fiber optics sensors, photonic crystal fibers, microstructure fabrication

## Abstract

A highly sensitive torsion sensor can be constructed by combining a twisted photonic crystal fiber with a liquid-filled waveguide in its air-hole cladding. The torsion sensitivity of this type of sensor is determined directly by the phase-matching conditions between the fiber core mode and the liquid waveguide mode, which can be improved by tuning the helicity (denoted by the initial twist rate, *α*_0_) of the twisted photonic crystal fiber. The enhancement mechanism of *α*_0_ on the sensitivity of the proposed torsion sensor is investigated theoretically, followed by experimental verifications, and a torsion sensitivity as high as 446 nm∙mm∙rad^−1^ can be obtained by tailoring these parameters. Experimental results show that the torsion sensitivity increases with *α*_0_ decreasing from 3.142 to 3.925 rad/mm, which are in consistence with that of the numerical predictions. The demonstrated torsion sensor is expected to contribute to the development of highly sensitive torsion-related photonic crystal fiber devices.

## 1. Introduction

Torsion measurements based on optical fibers have been applied to many fields including robotics, manufacture industry, and civil engineering. There are many different fiber structures that can be used for torsion sensing including long-period fiber gratings [1], optical fiber interferometers [2], fiber loop mirrors [2,3], and cascaded dissimilar fibers [4]. However, their inherent symmetry prevents discrimination of the rotational direction, unless extra pre-twist is applied on fiber sensors, which limits their practical applications. Thus, it is necessary to break the symmetry of fiber devices to construct sensitivity-improved torsion sensors while maintaining their direction discrimination. In recent years, helical structures have been implemented in optical fibers to demonstrate novel strain sensors [5], twist sensors [6,7,8], and tunable filters [9]. Some of these devices have been realized by employing photonic crystal fibers (PCFs) [10,11,12,13] and exhibited good torsion-sensing characteristics as well. Moreover, as is well-known, the unique arranged air holes in the cladding of PCFs provide flexible platforms for optical material infiltration and enable the creation of embedded satellite waveguides [14,15,16]. Therefore, ingenious directional coupling could be designed and tailored between the core mode and the embedded waveguide modes, and many interesting sensing properties can be obtained by tuning the effective refractive index (RI) of the waveguide modes. As such, with combining the helical structure and the liquid-filled waveguide in PCF cladding, sensors with superior sensing performances can be developed readily. Recently, we have proposed and demonstrated a torsion sensor based on a liquid-filled helical PCF (LFTPCF), which presents very high sensitivity and definite direction discrimination simultaneously for torsion measurement [17]. However, the response mechanism of the torsion sensitivity to initial twisting rate and the refractive index of the filled liquid has yet to be investigated.

In this work, we prepared a series of LFTPCFs with different initial twisting rate and refractive index of filled liquid to study the tunability of their torsion sensitivity. Through numerically simulating the phase-matching curves of the twisted PCF core mode and the liquid rod mode, we found that the initial twist rate *α*_0_ (rad/mm), the twisted radian per millimeter along the fiber in the fabricating process, had influence on the torsion sensitivity. The measurement results showed that the torsion sensitivities of these samples were determined by the initial twist rate of PCFs and the RI of the infiltrated liquid materials. As such, an optimal torsion sensitivity of 446 nm·mm·rad^−1^ is obtained, which is higher than that of previous reports [18,19,20].

## 2. Mode Analysis

**Figure 1:**
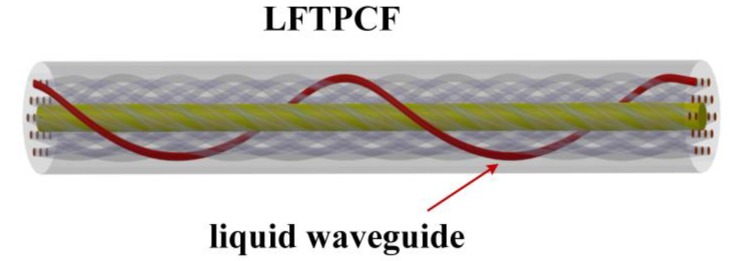
Schematic diagram of the LFTPCFs. The red path represented the liquid-filled air hole.

The proposed torsion sensor based on LFTPCFs was schematically illustrated in Figure 1. The PCF was first twisted with an initial twisting rate *α*_0_, and then one of the air holes in PCF cladding was selectively infiltrated by using liquid with a refractive index *n_l_*. To analyze the modal characteristics and predict the transmission spectra, the effective refractive index *n_eff_* of the guiding core mode and liquid rod mode in LFTPCFs with different fabrication parameters, *α*_0_ and *n_l_*, were simulated by combining the full vector finite element method with the Maxwell’s equations in a helicoidal coordinate system [21]. The PCF investigated in this work is ESM-12 (NKT Photonics), whose average air-hole diameter is 3.6 μm, hole-to-hole distance is 7.9 μm and cladding diameter is 125 μm. The filled liquid was standard refractive index (RI) liquid (Cargille Lab, Inc., Cedar Grove, NJ, USA) at around 1.484. Using the above parameters, the dispersion curves of *n_eff_* for core mode and liquid rod mode can be calculated simultaneously, and then the resonant wavelength at which the phase matching of mode coupling occurs can be obtained by finding out the crossing of the dispersion curves between the liquid waveguide mode and the fiber core mode.

**Figure 2:**
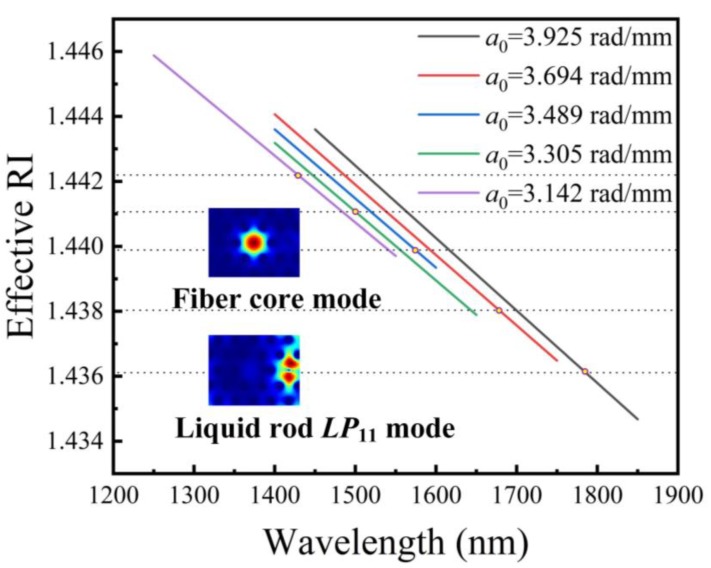
Dispersion curves of liquid waveguide *LP*_11_ modes in LFTPCFs with different *α*_0_. The yellow points identify phase-matching points between the core mode and the liquid waveguide *LP*_11_ mode under various *α*_0_, respectively.

When *α*_0_ was set to be 3.142, 3.305, 3.489, 3.694, 3.925 rad/mm, and *n_l_* = 1.484 the dispersion curves of liquid rod and the phase-matching point were calculated and plotted in Figure 2 respectively. But for the proposed sensor, the dispersion curve of fiber core mode is slightly different for each α_0_ and is not presented for clarity. The crossing points (namely, phase-matching points) of these two curves are marked by the yellow circles in Figure 2, respectively. It is worth to note that *n_eff_* declines while *α*_0_ decreases at a certain wavelength. However, the resonant wavelength that corresponds to the phase-matching point shifts toward shorter wavelengths because of the decrease in *α*_0_. It should also be noted that the liquid rod supports a few high-order modes for each case of *α*_0_. To clearly discuss the tendency in phase-matching points, only one typical *LP*_11_ mode is plotted in Figure 2. Mode profiles of the fiber core mode and the liquid rod *LP*_11_ mode of the sample with *α*_0_ = 3.142 rad/mm can be seen as the inset of Figure 2.

**Figure 3:**
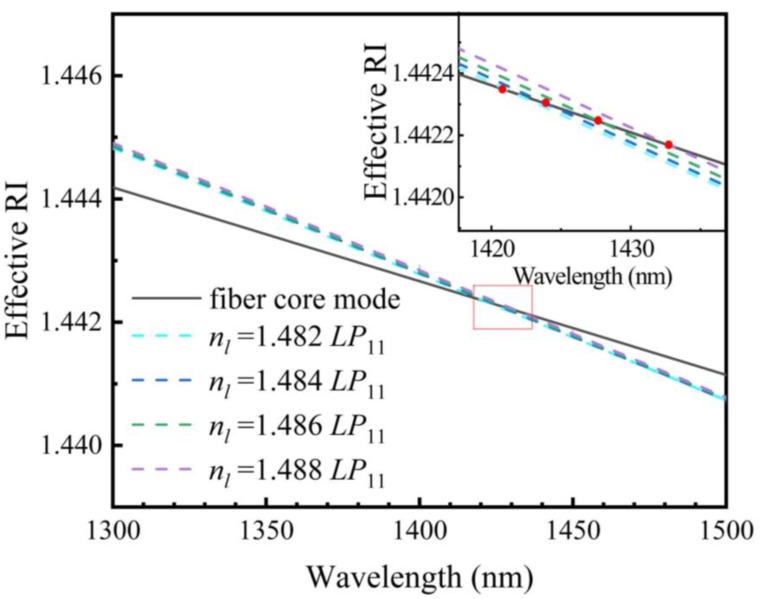
Dispersion curves of liquid waveguide *LP*_11_ modes in LFTPCFs with different RI liquids.

Meanwhile, the mode coupling characteristics of LFTPCFs with different RI liquids are also studied via numerical simulation. The calculated dispersion curves of the fundamental core mode and the rod mode *LP*_11_ are plotted in Figure 3, where one can see that both the effective modal RIs of the fiber core mode and the rod mode decreases with wavelengths increasing. At the same time, *n_eff_* of the liquid waveguide is raised obviously with *n_l_* increases, as can be seen clearly in the inset of Figure 3, which results in the red shift of the phase-matching point that corresponds to the coupling wavelength.

Above mentioned simulation results reveal that the resonant wavelengths can be improved by decreasing *α*_0_ or decreasing *n_l_*. The coupling between the higher modes of the helical liquid waveguide and the fundamental mode of fiber core can bring some new effects for torsion sensitivity. Meanwhile, the coupling efficiency and the measuring wavelength should be taken into consideration, which are closely related to engineering applications. The further investigation is under way.

## 3. Experiment and Discussion

**Figure 4:**
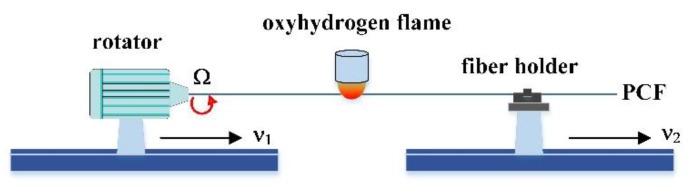
Schematic diagram of twisted photonic crystal fibers (PCF) by use of an oxyhydrogen flame.

**Figure 5:**
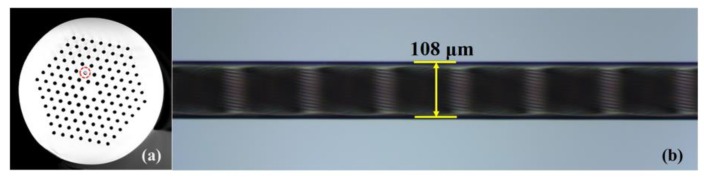
(**a**) Cross-section of the LFTPCF where one hole was filled with a standard refractive index (RI) liquid. The hole was outlined with a red circles. (**b**) Side-view microscope images of the LFTPCFs.

The proposed fiber sensors can be fabricated in two steps. A permanent helical structured PCF is first induced by simultaneously twisting and translating the normal PCF under an oxyhydrogen flame heating process as shown in Figure 4. Then, one of the air holes located in the second ring of the air hole cladding of the twisted PCF is filled with standard RI liquid (as shown in Figure 5) via a femtosecond-laser-assisted method [22]. Detailed fabrication process of the LFTPCFs has been demonstrated elsewhere [17]. In order to ensure repeatability of this heating process, the flow rate of hydrogen and oxygen and the translation stages were controlled precisely by computer. It can be observed and measured that under the same condition of oxyhydrogen flame heating, the air hole sizes and fiber diameters of the twisted PCFs are consistency.

The location of liquid-filled hole mainly determines the coupling length of the proposed structure, and the liquid RI determines the highest order of guiding modes that can be propagated in the liquid waveguide. In our experiment, standard liquid (Cargille Lab, Inc., Cedar Grove, NJ, USA ) with RI at around 1.484 is used, which means that the highest order of guiding mode in the liquid waveguide of our sample is *LP*_11_-like mode. As one of the hexagonally distributed air-holes located at vertex of the second ring is infiltrated, the coupling length between liquid waveguide *LP*_11_-like mode and PCF core *LP*_01_ mode is calculated to be about 30 mm [14,23]. This length is suitable for the experimental realization because it provides an appropriate tolerance of the infiltration length.

**Figure 6:**
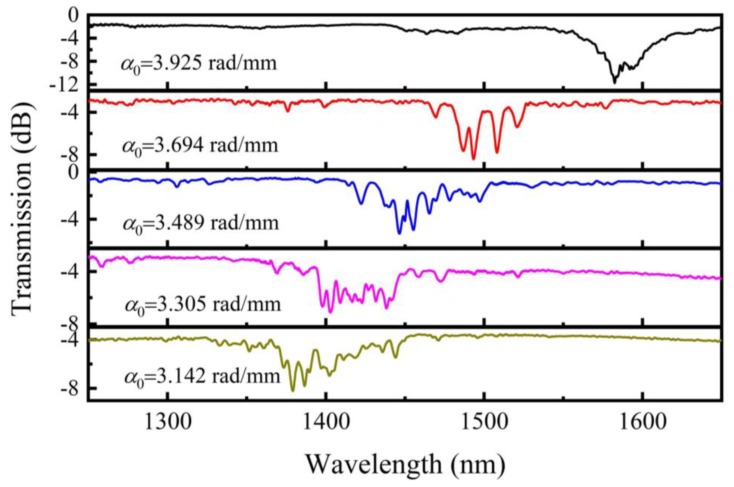
Transmission spectra of LFTPCFs with *α*_0_ varying from 3.142 to 3.925 rad/mm.

LFTPCF samples with a constant RI of 1.484 but different *α*_0_ (*α*_0_ = 3.925, 3.694, 3.489, 3.305 and 3.142 rad/mm) are prepared. The Figure 6 clearly shows the transmission spectra of these samples. The resonant dip in the transmission spectra locates toward shorter wavelengths as *α*_0_ decreases, which is consistent with the simulation result. The complicated structure of the resonant dip can be attributed to the optical degeneracy mode broken in a helical PCF. As the twisted PCF is selectively infiltrated with standard RI liquid, the liquid rod forms a waveguide helically wrapping around the PCF core as shown in Figure 1. As a result, the light of PCF core modes can be coupled into the helical waveguide mode when the effect RI curves are close to each other enough [24], resulting in some resonant dips in the transmission spectrum. It is noted that, the degeneracy within the *LP_lm_* mode group is broken in the helical reference frame, which will result in many resonant dips in the transmission spectra [25].

**Figure 7:**
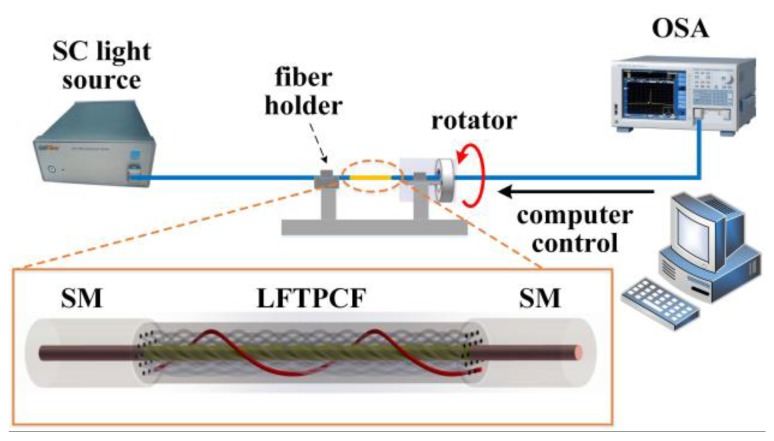
A schematic of the torsion test; inset is the structure diagram of the LFTPCF sensor.

In the torsion test system (Figure 7), LFTPCF sample was fixed between a rotator and a fiber holder. The distance between the two fiber holders (*L*) was 90 mm. The rotator can be rotated in clockwise (+) or counterclockwise (−) directions that are the same or opposite to the direction of PCF twisting, respectively. The two ends of the fiber are connected to a broad band ASE light source (wavelength range 1250–1650 nm, FL-ASE, FiberLake, Shenzhen, China) and an optical spectrum analyzer (OSA) (resolution 0.5 nm, scan speed 20 nm/s, AQ6370C, Yokogawa, Tokyo, Japan), respectively, to measure the transmission spectra. The applied twist rate *α* (rad/mm), the twisted radian per millimeter along the fiber in the torsion test process, can be estimated via the following relation, *α* = *θ*/*L*, where *θ* is the angle of rotation that can be varied from 0 to ±4π/3 rad in an interval of π/6 rad. Larger rotation angles have yet to be applied to avoid damaging the fiber in the torsion test.

For torsion test, the resonant dips can be traced while the torsion is applied. The torsion sensitivity *S_T_* is obtained by fitting the relation curve between *α* and the wavelength shift Δ*λ*. According to the simulation, it can be known that *n_eff_* can be affected by *α*_0_ and *n_l_*, which means that *S_T_* of the fabricated device can also be impacted by these two key factors. The value of *α*_0_ represents the helicity of the twisted PCF, and can be adjusted during the first fabrication step in experiment. The value of *n_l_* is the RI of infiltrated liquid material, and can be changed in the second preparation step.

**Figure 8:**
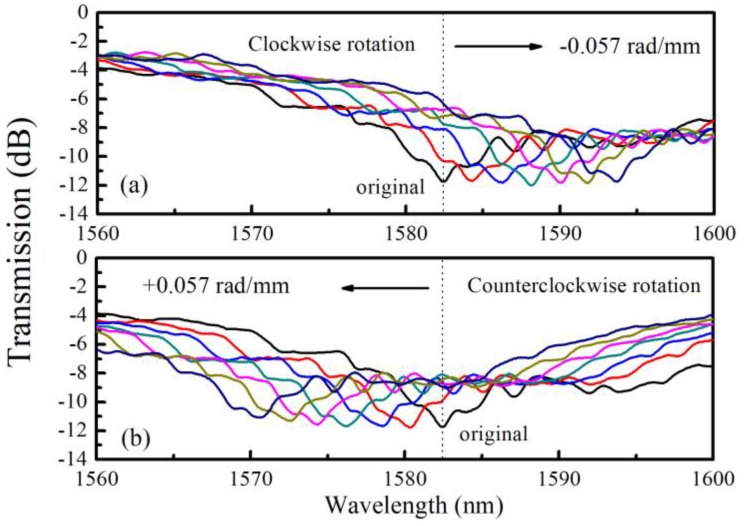
The resonant dip shift of the LFTPCF (*α*_0_ = 3.925 rad/mm) under an applied torsion in the range of −0.057 to +0.057 rad/mm in step of 0.0095 rad/mm.

**Figure 9:**
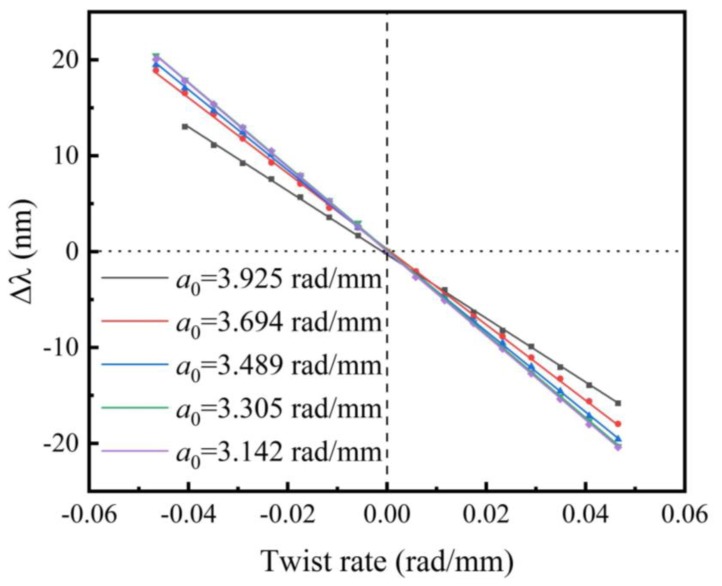
The linear fitting between the applied twist rate and wavelength shifts for the LFTPCFs with various *α*_0_.

The resonant dip shift is sensitive to torsion, and the shift directions for clockwise and counterclockwise can be distinguished because of the helical structure of the liquid rod waveguide [17]. By changing *α*, the transmission spectra of sample with *α*_0_ = 3.925 rad/mm, for example, are recorded as shown in Figure 8. It is obvious that the dip shifts to the opposite direction as the sensor is rotated oppositely. In order to calculate the sensitivity, we track the dip wavelength shift of the deepest one as the reference and plot them in Figure 9. Linear fitting of these data reveals that the resonant wavelength shift linearly with respect to *α*. As such, the torsion sensitivities *S_T_* are calculated and listed in Table 1. As *α*_0_ decreases, the torsion sensitivity of the samples increases more than 100 nm∙mm∙rad^−1^ from 333 to 439 nm∙mm∙rad^−1^. The changing trend of *S_T_* is same as that of *n_eff_*. When the value of *n_eff_* increases, the *S_T_* improves as well.

**Figure 10:**
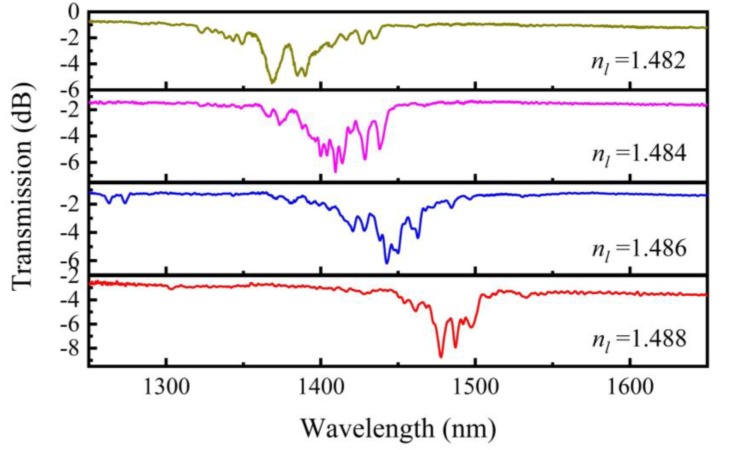
Transmission spectra of LFTPCFs filled with standard RI liquid as well as RIs that varied from 1.482 to 1.488 in intervals of 0.002.

To study the effect of liquid RI on torsion sensitivity, samples with an initial twist rate of 3.142 rad/mm are fabricated to be filled with different liquid materials. Subsequently standard RI liquids (*n_l_*) with 1.482, 1.484, 1.486, and 1.488 are filled in these samples, respectively. The transmission spectra of these samples are measured at room temperature as shown in Figure 10. It is easy to find that the resonant dip red shifts with *n_l_* increasing in a wavelength range from 1300 to 1600 nm.

**Figure 11:**
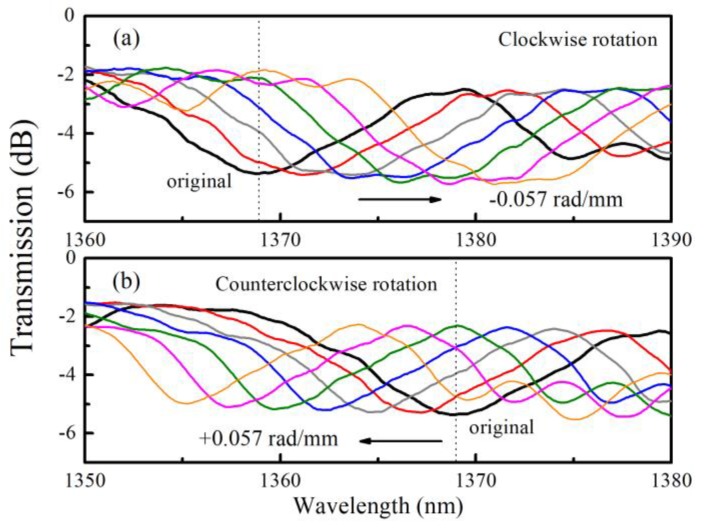
The resonant dip shift of the LFTPCF (*n_l_* = 1.482) under an applied torsion in the range of −0.057 to +0.057 rad/mm in step of 0.0095 rad/mm.

**Figure 12:**
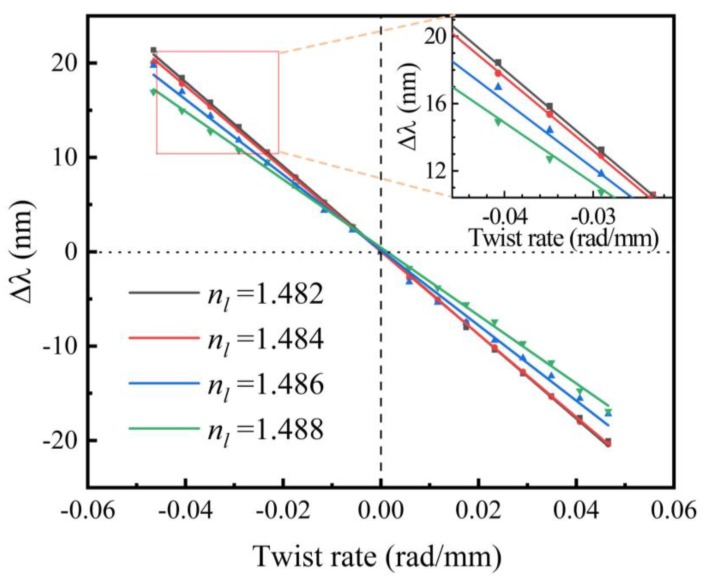
Linear fitting between the applied twist rate and wavelength shifts for the LFTPCFs filled with standard liquid with various RI.

For instance, the spectral responses of sample with *n_l_* = 1.482 for different angles of rotation are plotted in Figure 11. The wavelength variations of the resonant dips are plotted versus *α*, as illustrated in Figure 12. The torsion sensitivities of these samples are calculated through linear fitting and listed in Table 2. As *n_l_* decreases, the torsion sensitivity of the samples increases from 360 to 446 nm∙mm∙rad^−1^. According to the results of simulation, the decrease of *n_l_* can lead to an increase of *n_eff_*. It is shown that the changing trend of *S_T_* is same as that of *n_eff_*.

Through the torsion test experiment and the simulation, the results show that the torsion sensitivities of these samples are related to the values of *n_eff_*. The sample with larger *n_eff_* has higher *S_T_*. According to the dispersion property of fiber core, the resonant wavelength should locate at shorter wavelength in order to obtain larger *n_eff_*. The control of resonant wavelength can be achieve in two ways: One is to change the initial twist rate of twisted PCF, and the other is to vary RI liquid that is infiltrated in twisted PCF. That is, the torsion sensitivity of this kind of fiber sensor can be optimized through these methods. The reason for using standard IR liquids to fill the PCF is simply because the chromatic dispersion of these liquids are known, which is easy to quantitative simulation and analysis. On the other hand, once a non-standard RI liquid is filled in the twisted PCF, the torsion sensitivity will be enhanced if the effective RI corresponding to the resonant wavelength is larger compared to the case of using a standard RI liquid, and vice versa.

As shown in Table 2, the optimum torsion sensitivity of LFTPCFs is 446 nm∙mm∙rad^−1^. A variety of previously reported fiber optic torsion sensors that employed different special fibers and structures are presented in Table 3 for comparison, where the achieved torsion sensitivity of the proposed sensor is far higher than that of the torsion sensor reported so far.

## 4. Conclusions

In conclusion, we proposed a series torsion sensors based on twisted photonic crystal fibers with an embedded helical liquid waveguide in the cladding. Because of the directional coupling between the fiber core mode and the liquid waveguide modes, resonant dips appear in the transmission spectrum from 1250 to 1650 nm. The mechanism was analyzed theoretically. The resonant wavelength and the effective refractive index corresponding to phase-matching point can shift while the initial twist rate or RI of infiltrated liquid changing. The torsion tests of the samples were made experimentally in order to investigate the impact of initial twist rate on torsion sensitivities. It is noted that torsion sensitivity is associated with the effective refractive index. The results revealed a method to prepare a new sensor with high torsion sensitivity. The optimal torsion sensitivity was ~446 nm∙mm∙rad^−1^. The helical PCF with an embedded waveguide can be a valuable torsion sensor.

## Figures and Tables

**Table 1:** Torsion sensitivities (*S_T_*) of the LFTPCFs with different twist periods.

***α*_0_ (rad/mm)**	3.925	3.694	3.489	3.305	3.142
***S_T_* (nm∙mm∙rad^−1^)**	333	395	420	437	439

**Table 2:** Torsion sensitivities (ST) of the LFTPCFs with different standard RI liquid.

***n_l_***	1.482	1.484	1.486	1.488
***S_T_* (nm∙mm∙rad^−1^)**	446	439	399	360

**Table 3:** Torsion sensitivities of several fiber sensors.

Fiber Sensor	Torsion Sensitivity (nm·mm·rad−1)	References
LFTPCF	446	
Helical PCF	56	[19]
CLPGs	210	[26]
Helical MZI	261	[20]
Multicore fiber MZI	118	[27]
Conventional LPFGs	23	[18]
